# Intrasegmental recombination as an evolutionary force of Lassa fever virus

**DOI:** 10.3389/fmicb.2024.1411537

**Published:** 2024-05-20

**Authors:** Cheng-Qiang He, Chao Kong, Mei He, Guan-Xiang Chen, Shu-Min Liu, Nai-Zheng Ding

**Affiliations:** Shandong Provincial Key Laboratory of Animal Resistance Biology, College of Life Science, Shandong Normal University, Jinan, China

**Keywords:** Lassa fever, LASV, homologous recombinant, virus evolution, negative-stranded RNA virus

## Abstract

Lassa fever (LF), caused by Lassa virus (LASV), is one of the most dangerous diseases to public health. Homologous recombination (HR) is a basic genetic power driving biological evolution. However, as a negative-stranded RNA virus, it is unknown whether HR occurs between LASVs and its influence on the outbreak of LF. In this study, after analyzing 575 S and 433 L segments of LASV collected in Africa, we found that LASV can achieve HR in both of its segments. Interestingly, although the length of S segment is less than half of the L segment, the proportion of LASVs with S recombinants is significantly higher than that with L recombinants. These results suggest that HR may be a feature of LASV, which can be set by natural selection to produce beneficial or eliminate harmful mutations for the virus, so it plays a role in LASV evolution during the outbreak of LF.

## Introduction

Lassa fever (LF) is a type of viral hemorrhagic fever with high mortality, which has been listed by the World Health Organization (WHO) as one of the diseases posing the greatest risk to public health.^1^ It is estimated that LF causes tens of thousands of hospitalizations and thousands of deaths every year. Case fatality rates among hospitalized LF patients can be up to 50%, although numerous sub-clinical infections may also occur (Troup et al., 1970; McCormick and Fisher-Hoch, 2002). Its pathogen, Lassa virus (LASV), is classified as (BSL)-4 agent, which causes endemic diseases in much of West Africa, primarily including Sierra Leone, Guinea, Liberia, and Nigeria (Andersen et al., 2015). The rodent *Mastomys natalensis* functions as a reservoir and maintains persistent infections of the virus (Lecompte et al., 2006). Although most patients are infected by exposure to excreta from this animal, human-to-human transmission has also been reported (McCormick and Fisher-Hoch, 2002). Given its potential to cause a public health emergency and the absence of efficacious drugs and vaccines, the WHO Research and Development (R&D) Blueprint Committee suggested that there is an urgent need for accelerated R&D for the virus (Mehand et al., 2018).

LASV belongs to the *Mammarenavirus* genus of the Arenaviridae family (Pennington and Lee, 2022). Its genome consists of two segments of negative-sense single-stranded RNA, namely S and L segments (Torriani et al., 2017). Their lengths are approximately 3,400 and 7,300 nucleotides, respectively. They ambisense strategy is used to encode four proteins, namely, large (L) protein (RNA-dependent RNA polymerase, RdRp), zinc-binding (Z) protein, nucleoprotein (NP), and glycoprotein complex (GPC) (Garry, 2022). The L protein is encoded at the 3′ end of the L segment in the genome-complementary sense, whereas the Z protein is encoded at the 5′ end of the L RNA in the genomic sense. In a similar fashion, the *np* gene encodes NP at the 3′ end of the S RNA, whereas GPC is encoded at the 5′ end of the S RNA. Of them, glycoprotein precursor undergoes proteolytic cleavage to yield three subunits, namely, GP1, GP2, and the stable signal peptide (SSP) (Pennington and Lee, 2022). SSP/GP1/GP2 lies on the virion surface and mediates host cell attachment and membrane fusion and entry (Hastie and Saphire, 2018). As the antigen on the viral surface, GP1 is also the response target of protective antibody of host and becomes a focus for vaccine design efforts (Sommerstein et al., 2015; Robinson et al., 2016).

As an RNA virus, LASV has wide genetic diversity. Phylogenetic analyses showed that LASVs have differentiated into seven genotype lineages associated with geographical locations (lineages I, II, III, IV, V, VI, and VII). Sequence diversity is as high as 24.6% between lineages (Bowen et al., 2000). Moreover, each lineage may also include different sublineages (Oloniniyi et al., 2018). The LASV circulating in Nigeria has the most extensive genetic diversity, including lineages I, II, and III, whereas strains from Guinea, Sierra Leone, and Liberia seemed to be more closely related and belong exclusively to lineage IV (Fichet-Calvet et al., 2016). Lineage V is mainly found in Mali (Gunther et al., 2001). Lineage VII was reported from Togo in 2016 (Whitmer et al., 2018). The regionalization of genetic diversity may be the consequence of the adaptation of the virus to the local niches. Understanding the molecular mechanism of genetic diversity is important for controlling LASV.

Due to the lack of proofreading function, RdRp of LASV can result in a high mutation rate in progeny virus genome during replication. The evolution rate of LASV was as high as 10^−3^/site/year (Andersen et al., 2015). However, high mutation rates are generally considered deleterious for RNA virus due to its replication mode, which causes a continuous decrease in the mean fitness of its population (Chao, 1990). As a form of similar to sexual reproduction (Simon-Loriere and Holmes, 2011), Genetic recombination can compensate for the adverse effects of the high mutation rate (Bodine et al., 1990; Naito and Pawlowska, 2016). Recombination in RNA viruses can be defined as an exchange of genetic material between at least two separate viral genomes. Two types of recombination are known as reassortment and homologous recombination (HR). The former is defined as the exchange of genomic segments between viruses in a segmented genome, while the latter involves the exchange of homologous regions of genome isolated by one or several breakpoints (Simon-Loriere and Holmes, 2011). HR also plays an important role in the evolution of some positive-stranded RNA viruses (Kirkegaard and Baltimore, 1986; Nagy and Simon, 1997). Virulent variants of some viruses have been generated by homologous recombination (Worobey et al., 1999; Anderson et al., 2000; Pita et al., 2001). Nevertheless, HR has been reported to be rare in negative-stranded RNA virus because of its genome packaged with nucleoproteins (Chare et al., 2003). Despite this, there is evidence of recombination in negative-stranded RNA viruses, such as influenza virus (He et al., 2008, 2019), Newcastle disease viruses (Zhang et al., 2010), rabies virus (Ding et al., 2017), Zaire ebolavirus (Wittmann et al., 2007), and severe fever with thrombocytopenia syndrome bunyavirus (He and Ding, 2012). So far, the occurrence and evolutionary influence of HR have not been known in LASV evolution, although several groups have attempted to find whether HR affects the evolution of this virus (Chare et al., 2003; Andersen et al., 2015).

In this study, we provide a series of significant evidence to show that HR has occurred in LASV. The recombination was found in both segments of LASV and could take place between viruses of the same or different lineages. Moreover, an LASV with recombinant S segment has become the dominant variant in response to 40% of LF cases in Nigeria. These results show that HR plays an important role in shaping LASV evolution and has the potential to result in a novel highly pathogenic LASV for human and/or host tropism change.

## Materials and methods

### Viruses and sequences

**Figure 1:**
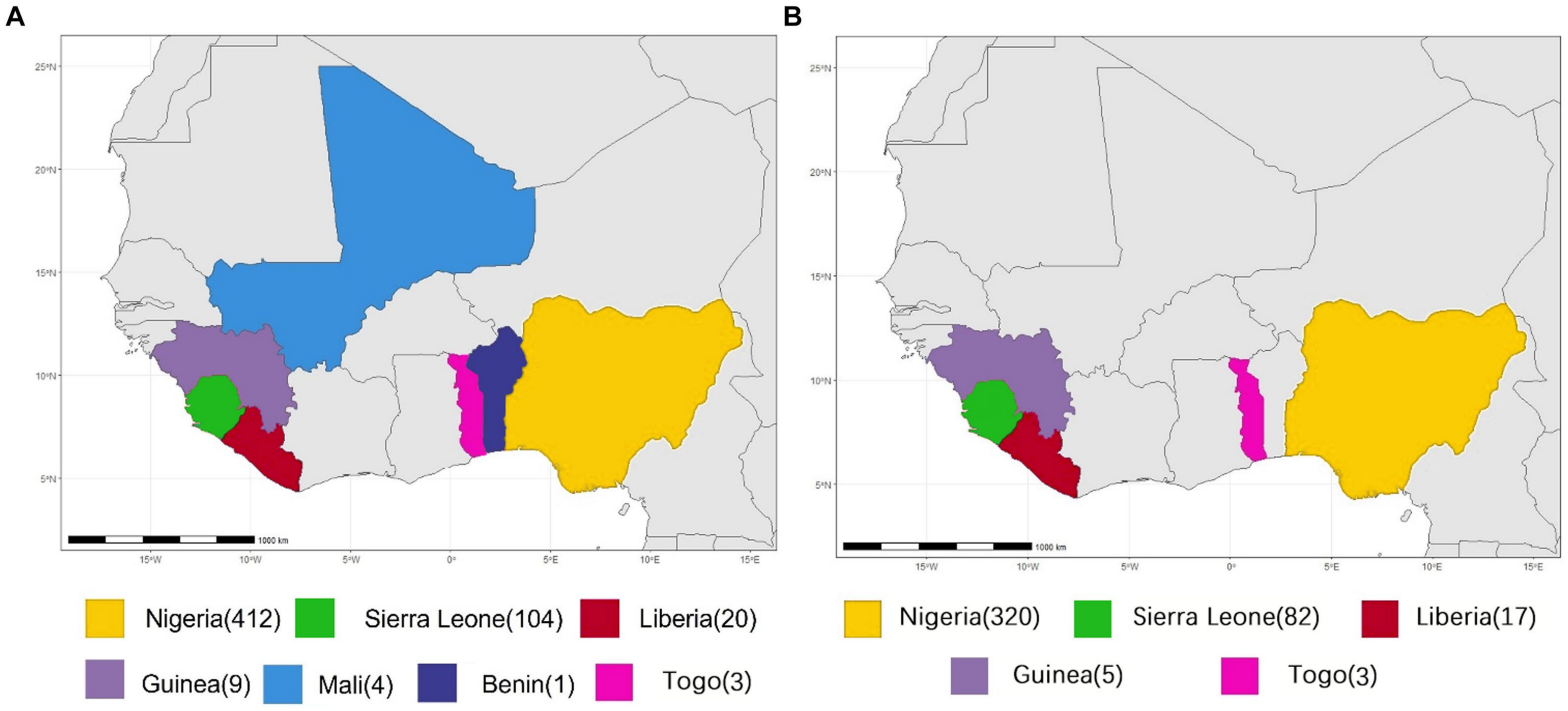
Geographical distribution of LASV samples for homologous recombination analysis. **(A)** S segment. **(B)** L segment.

All available complete LASV S and L segment sequences were downloaded from GenBank. Most of these viruses are sequenced by the next-generation sequencing method. Since low coverage and co-infection might result in ambiguous bases, those sequences containing many “N” bases were considered as low quality and discarded in order to avoid unreliable recombination results as much as possible, Finally, 575 S and 433 L segments were used to analyze potential HR. Their geographical distributions are shown in Figure 1. These viruses were mainly collected from Nigeria, accounting for >70%.

### Reconstruction of phylogeny

These sequences were aligned with CLUSTAL W (Thompson et al., 1997). Nucleotide substitution models with the lowest Bayesian Information Criterion (BIC) scores are considered as the best substitution pattern. Phylogenetic histories were inferred using the Maximum-Likelihood (ML) or the Neighbor-Joining (NJ) methods, with the best nucleotide substitution model. The bootstrap method was used to assess the robustness of tree branches with 1,000 replicates. A branch with more than 70% bootstrap value was considered to have a recent common ancestor with robustness. Evolutionary analyses were conducted in MEGA11 (Tamura et al., 2021). Shimodaira-Hasegawa (SH) test method, which was implemented in TREE-PUZZLE program (Schmidt et al., 2002), was employed to test whether topology of phylogenetic trees was significantly different.

### Recombination analysis

The recombination analysis was performed referring to previous report (He et al., 2010). In brief, the recombination signals are visualized using the Simplot and Bootscan programs implemented in the Simplot software package (Lole et al., 1999). Employing the FindSites subprogram of Simplot, potential breakpoint was estimated at parsimonious region with the maximization of chi-square values (χ^2^) of the information sites. Fisher’s exact test was used to statistically analyze the difference of information sites on both sides of the breakpoints. At the same time, the sequence alignment data were examined for potential recombination events using the RDP software package (Martin et al., 2005). If a recombinant signal can be detected by at least two recombination detection methods, the signal is considered robust.

To demonstrate the presence of recombination, the gold-standard approach is a set of incongruent phylogenetic trees inferred from the different genomic regions of the putative recombinant viruses (Chare et al., 2003; Boni et al., 2010). The ML method implemented in MEGA 11 was used to reconstruct the phylogeny of different regions defined by the recombination breakpoints of the recombinants (Tamura et al., 2021). The difference in significance of the topology of these phylogenetic trees was confirmed by the SH test (Schmidt et al., 2002). If different regions have different phylogenetic histories, the recombination signs of these recombination groups are considered as robustness.

## Results and discussion

In this study, recombination was analyzed in the 575 S and 433 L segments. 193 S and 21 L segments were found to have significant recombination signals (Table 1 LASV strains with evidence for potential recombination). This means that more than one-third of the LASVs circulating in Africa might carry recombinant S segment. Among the 193 viruses, 5 were single isolates while the other 188 composed of 6 recombinant groups. The largest group consisted of 168 members. Interestingly, although the length of L segment is more than twice that of S segment, the recombinants found in L segment were significantly lower than in S segment, which were less than 5%. In addition, considering that HR might not be frequent in the virus, those isolates with the same recombination event should be descended from a common recombinant ancestor. These results suggested that HR is likely a trait of LASV that can be set by natural selection to create advantageous or purge deleterious mutations in this virus.

**Table 1:** LASV strains with evidence for potential recombination.

GenBank number	Recombinant	Num. of Sisters	Segment	Putative parents	Position of Breakpoints	Host	Country	Sequencing Technology
MH053560	ISTH_1150	0	S	ISTH_0543; ISTH_1250	2,907	Human	Nigeria	Illumina MiSeq
MK107957	IRR_030	0	S	IRR_027; 0167-ONDO	93, 556, 1,020, 1,218, 2,877, 3,004	Human	Nigeria	Illumina MiSeq
MK107947	IRR_003	0	S	IRR_005; ISTH_0621	1,412, 1,581	Human	Nigeria	Illumina MiSeq
MH887871	0169-ONDO	0	S	0106-ONDO; 0083-ONDO	2,151, 2,401, 3,067	Human	Nigeria	Illumina MiSeq
MK107970	IRR_004	0	S	IRR_002; ISTH_0543	3,018; 3,248	Human	Nigeria	Illumina MiSeq
OM735974	Barlie-00241	1	S	G1774-SLE; G1618-SLE	1,095; 2,124; 2,584; 2,939	*L. sikapusi*	Sierra Leone	Illumina
KM821877	G2906-SLE	2	S	G1774-SLE; G2392-SLE	2,515; 3,045	Human	Sierra Leone	Illumina
MK118000	IRR_073	3	S	ISTH2271-NIG; 0133-EDO	194; 3,001	Human	Nigeria	Illumina
MK107920	IRR_028	5	S	IRR_005; IRR_015	346; 559	Human	Nigeria	Illumina
MK107951	IRR_001	5	S	IRR_011; ISTH1096-NIG	688; 1,120; 2,778	Human	Nigeria	Illumina
KM822069	LASV966	167	S	0144-ONDO; 1,008-NIG	1,368; 2,169	Human	Nigeria	Illumina
MK117929	IRR_096	0	L	0192.2-ONDO; 0193-DELTA	2,819; 3,634	Human	Nigeria	Illumina
MK117903	IRR_052	18	L	IRR_071; Nig08-A37	378; 584	Human	Nigeria	Illumina
MH887906	LASV0169	0	L	IRR072; LASV0215	376; 740; 1,055; 6,425	Human	Nigeria	Illumina

### HR has influence on the evolution of S segment

S segment encodes GPC and NP proteins of LASV. NP oligomerizes and encapsidates genomic RNA to form a nucleocapsid (Young and Howard, 1983). Although the core structural function of NP oligomers is used to package, protect, and control access to the viral genome (Ruigrok et al., 2011; Reguera et al., 2014), NP also suppresses host type I interferon response (Huang et al., 2018), suggesting that it plays a role in adapting to the innate immunity of the host. As the extracellular spike, GP1 of the surface glycoprotein mediates the receptor (α-dystroglycan, an extracellular protein) recognition and viral entry into a host cell and bears the strong immune selection pressure from the host (Burri et al., 2012; Hastie and Saphire, 2018). Therefore, rapid evolution of S segment may be the inherent requirement for LASV to adapt to selection pressure from immunity of the host. In the samples we analyzed, more than 30% of viruses contained recombinant S segment. Interestingly, LASV966 recombinant family has caused more than 45% cases of LF in Nigeria.

**Figure 2:**
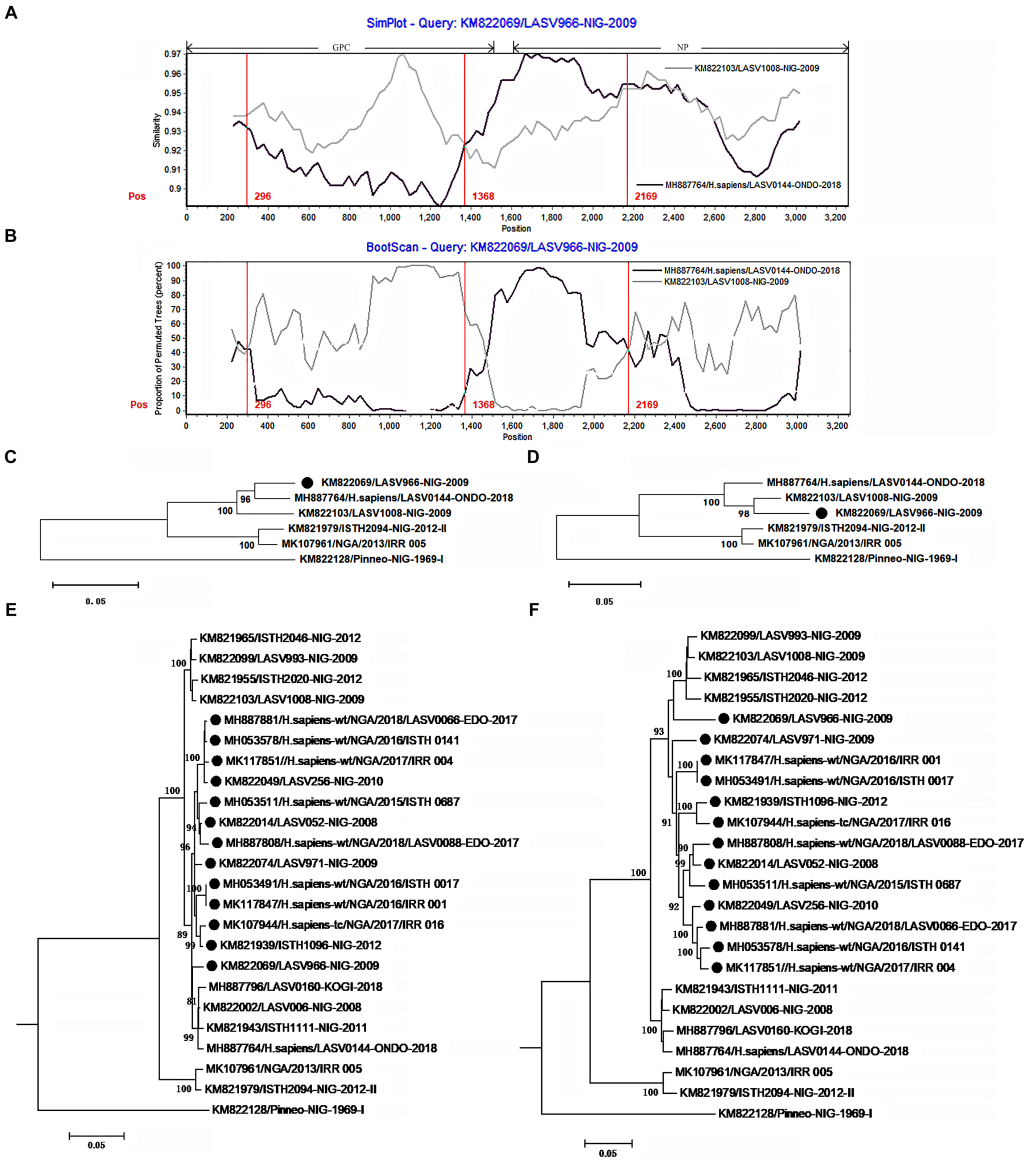
The evidence for recombination in the S segment of LASV966 and its sisters circulating in Nigeria. **(A)** Comparison of S segments of the recombinant LASV966 and its putative parents LASV1008 and LASV0144. LASV966 is used as the query. The *y*-axis gives the percentage of identity between LASV966 and its two parents. Different colors on the bold line represent the distribution of genes in the S segment. **(B)** Bootscan plot of S segment sequences of LASV966 and its putative parents. The *y*-axis gives the percentage of permutated trees using a sliding window. The vertical lines show the recombination breakpoints with the maximum χ^2^ of information sites. **(C,D)** Phylogenic trees inferred from different regions of S segment. **(C)** The phylogenic tree inferred from the regions 1 to 300 and 1,368 to 2,169. **(D)** The phylogenic tree inferred from the regions 300–1,368 and 2,169–3,025. **(E,F)** Incongruent phylogenetic histories of the S recombinant lineage. **(E)** The phylogenetic history of the 13 S recombinants inferred from the regions 1 to 300 and 1,368 to 2,169. **(F)** The phylogenetic history of the 13 S recombinants inferred from the regions 300–1,368 and 2,169–3,025. The S recombinants are marked with “●”.

The isolate LASV966 was collected from a patient in 2009 in Nigeria (Andersen et al., 2015). Its genome was sequenced using Illumina method and assembled *de novo* through Trinity v. r2011-11-26 and Novoalign v. v2.08. Previous study showed that its genome was sequenced with high quality (Andersen et al., 2015). This study found that LASV966 and its sister branch consisting of more than 160 members contained the same recombinant S segment (Figure 2; Supplementary Figure S1).

With the help of Simplot program, the S segment sequences of LASV966 and its parent lineage representatives LASV0144 and LASV1008 were compared (Figure 2A). The translation starting site of GPC is defined as nucleotide 1. Two crossover sites were found around the positions 1,368 and 2,169. The recombinant LASV966 and the putative minor parent LASV0144 have higher sequence similarity between the two crossover sites. However, outside of the two crossover sites, the sequence similarity between LASV966 and LASV1008 was higher than that of LASV0144. Bootscan analysis showed that there might be three crossover sites on the S segment of LASV966, and the new crossover sites were located near position 300 (Figure 2B). The maximum χ^2^ values of the three putative breakpoints were 0.6, 20.08, and 13.09, respectively. It was found that the first breakpoint had no statistical significance support (*p* > 0.05 of Fisher’s exact test). For the second and third breakpoints, their information sites had statistically significant differences (*p* < 0.0001 and *p* < 0.0005, respectively). Employing the recombination detection program (RDP 4.0), six recombination detection methods, RDP (*p* < 0.005), GENECONV (*p* < 0.05), BootScan (*p* < 0.05), Maxchi (*p* < 0.0001), Chimera (*p* < 0.005), and 3Seq (*p* < 0.0001) found significant evidence of recombination. The most compelling evidence for recombination was the occurrence of incongruent phylogenetic trees in different genomic regions (Nelson and Holmes, 2007), which was found in LASV966 S segment (Figures 2C,D). These results suggested that S segment of LASV966 had multi-origin. The most likely parent was sought in GenBank using the basic local alignment search tool (BLAST), but putative parent with 100% sequence identity could not be found, indicating that S segment of LASV966 might originated from a recombinant ancestor that had experienced multiple recombination events before 2009.

Running RDP, we scanned the sequence alignment data of S segments and found another 167 recombinants identical to LASV966, and they accounted for more than 40% (168 of 412) of the Nigerian LASV in our sample. These viruses have been circulating in Nigeria in 2008, indicating that the group with the recombinant S segment should have become the dominant pathogen responsible for LF in this country.

To determine these recombinants, 13 representatives were used to reconstruct their phylogenetic histories (Figures 2E,F). In the phylogenetic tree inferred from the regions 1–300 and 1,368–2,169, they were clustered into the LASV0144 lineage. However, in the phylogenetic tree inferred from the regions 300–1,367 and 2,169–3,052, the 13 isolates and the LASV1008 lineage constituted a mono-phylogenetic group. Moreover, there was a statistically significant discrepancy in the topology of the two trees (Shimodaira–Hasegawa test, *p* < 0.05), which also constituted a powerful evidence for the recombination event. It was also noticed that 147 of the 168 apparent S recombinants had the signal of multiple origins (Supplementary Figure S1).

In addition to the LASV966 family, another 19 S recombinants have also been found in the LASV circulating in Nigeria (Supplementary Figures S2–S9). Of them, 5 recombinants were single, and the other 14 formed three groups. Moreover, except for the recombinant ISTH_1150 (Supplementary Figure S5), there are at least two potential recombination breakpoints in these S recombinants.

In S segment of the isolate IRR 030, there were up to six obvious recombination breakpoints (Supplementary Figure S2). This virus has been purified by cell culture, and virus purification was performed by cell culture to obtain a high-titer viral stock which was suitable for subsequent experiments. This method ensures the removal of contaminants from other viruses, thereby providing a purified virus sample for accurate characterization of viral properties. In addition, there is an N base in the nucleotide sequence of its segment S during Illumina MiSeq sequencing. Moreover, there is no recombination signal in its segment L. These results suggested that these recombination signals should be more likely natural, although further sequencing verification is necessary.

It was noticed that these recombination events mainly occurred between viruses within the same lineage. This is understandable because viruses within lineages have a higher chance of meeting in the same host than viruses outside lineages. Interestingly, recombination might take place between different lineages (Supplementary Figure S3). IRR 003 has also been purified by cell culture (Ehichioya et al., 2019). In its S segment, there are two significant recombination breakpoints located at the 3′ end of GPC and NP genes. In S segment of IRR 003, most of the genetic material were inherited from the lineage II, but there were approximately 170 bases inherited from lineage III.

In this study, the sample also included 104 isolates from Sierra Leone. Of them, five recombinants were found with two different recombinant S segments. Three human isolates contain one of them (Supplementary Figure S10), while two rodent isolates contain the other (Supplementary Figure S11). All these five viruses had been sequenced in high quality, and there were no ambiguous bases in their S segment.

The three human isolates with the recombinant *np* gene were collected in 2011, 2012, and 2013, respectively. In addition, no putative parent strains can be found in the GenBank database, which are completely consistent with their nucleotide sequences. These results indicated that the recombination event should have occurred before 2011. Although the recombinant did not become the dominant strain of Sierra Leone, it repeatedly infected humans, indicating that it could have been widely circulating in rodents of this country.

### HR also affects the evolution of L segment

**Figure 3:**
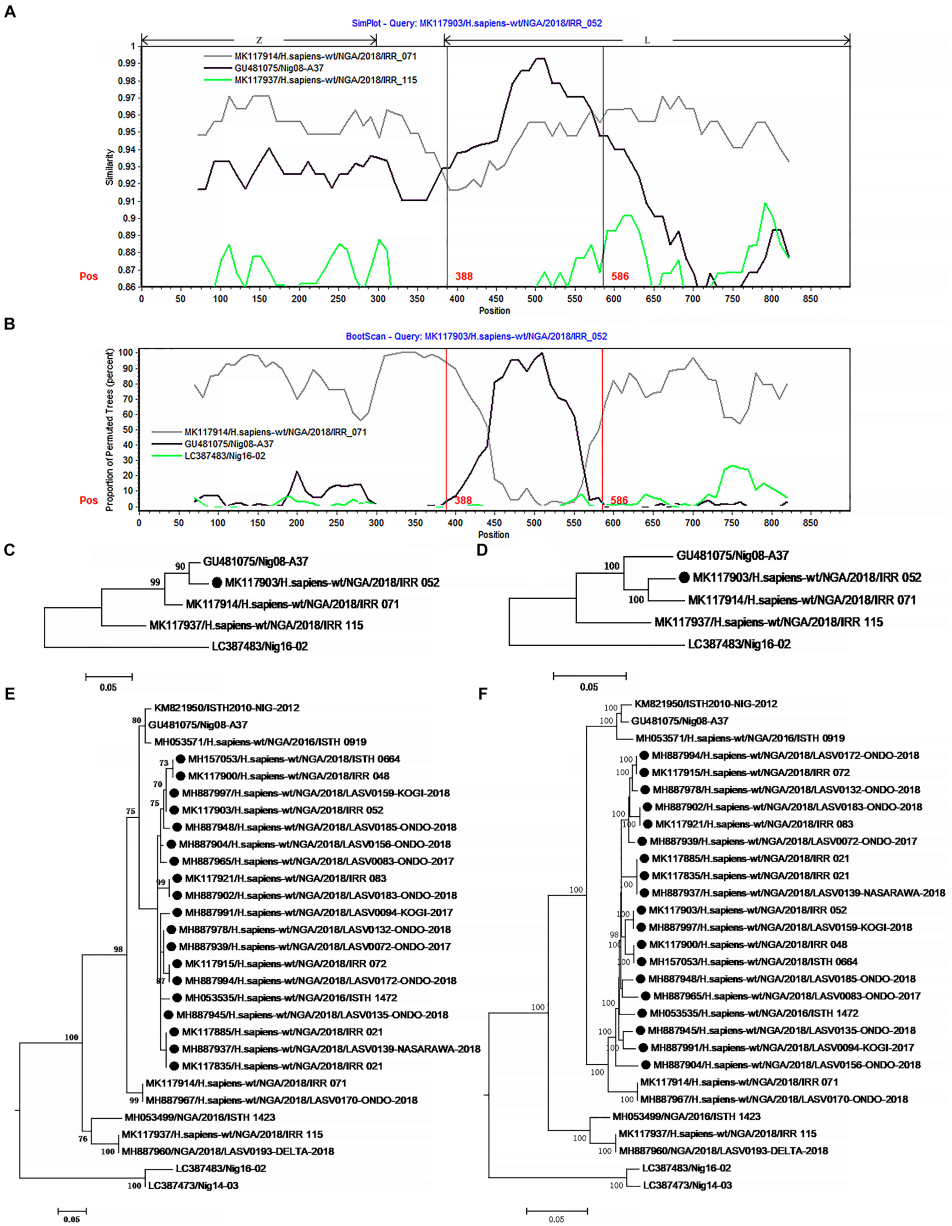
The evidence for recombination in the L segment of IRR 052 and its sisters circulating in Nigeria. **(A)** Sequence similarity plot of L segment (positions 1–900) of IRR 052 and its putative parents IRR 071 and Nig08-A37. IRR 052 is used as the query. The y-axis indicates the percentage of similarity between IRR 052 and its parents. Different colors on the bold line represent the different genes in the L segment. **(B)** Bootscan plot of L segment sequences of IRR 052 and its putative parents. The y-axis gives the percentage of permutated trees. The vertical lines indicate the recombination breakpoints. **(C,D)** are the phylogenic trees inferred from different regions of L segment. **(C)** The phylogenic tree inferred from the region 388–586. **(D)** The phylogenic tree inferred from the regions1-388 + 586–6,500. **(E,F)** Incongruent phylogenetic histories of the L recombinant lineage. **(E)** The phylogenetic history of the 19 L recombinants inferred from the fragment 388–586. **(F)** The phylogenetic history of the 19 L recombinants inferred from the region 586–6,500. The recombinant isolates are marked with “●”.

L segment encodes Z and L proteins. The Z protein coordinates the packaging, budding of infectious particles, and virus egress (Urata and Yasuda, 2012). L protein is a multifunctional enzyme with RNA-dependent RNA polymerase, host-cap-snatching endonuclease, and regulatory domains (Vieth et al., 2004; Brunotte et al., 2011; Vogel et al., 2019). As the inner proteins, these two proteins may have undergone less natural selection pressure. Therefore, the selection orientation of recombinant L might be relatively lower. In fact, only 21 isolates were found to carry recombinant L segment among the 433 viruses analyzed in this study (Figure 3; Supplementary Figures S12, S13). The rate of recombinant is less than 5% of the sample. This is much lower than the 33% recombinant ratio of the S segment. Of the 21 L recombinants, 19 might originate from the same common ancestor which underwent the HR. In addition, the single sequence of the recombinant IRR 0096 contains many ambiguous bases which are evenly distributed in its L segment.

The 19 L recombinants shared more than 96.3% sequence similarity with each other. For recombination analysis, IRR052 was used as the recombinant representative strain. Two isolates, IRR071 and Nig08-A37, could be supposed as the putative parents of the recombinant. Combined with the results of the χ^2^ analysis and Fisher’s exact test, two breakpoints were located at the positions 388 and 586 with maximum χ^2^ values (5.4 and 9.6, respectively) when the start codon of the Z gene was defined as the first nucleotide. These two breakpoints fall in the 3 ‘end of the L gene (Figure 3A). Fisher’s exact test showed that the information sites on both sides of these two breakpoints were significantly different (*p* < 0.05 and *p* < 0.01, respectively). We compared the similarity between the first 900 nucleotides of the L segment of IRR052 and its two putative parents (Figure 3A). Between the two breakpoints, IRR052 shared 96.9% sequence identity with the putative minor parent Nig08-A37 (versus 93.4% with IRR071). However, before and after these two breakpoints, IRR052 only shared 90.9% similarity with Nig08-A37 (versus 95.4 with IRR071). Bootscan result supported the hypothesis that IRR052 was descended from the two lineages, IRR071 and Nig08-A37 (Figure 3B). The recombination signal was also recognized by RDP, and its five programs, namely, GENECONV (*p* < 0.05), BootScan (*p* < 0.01), Maxchi (*p* < 0.01), Chimera (*p* < 0.05), and 3Seq (*p* < 0.05) gave statistically significant evidence of recombination. Phylogenetic reconstruction showed that IRR052 and Nig08-A37 constituted a monophylogenetic group with 90% bootstrap value (Figure 3C) between the two breakpoints. On the contrary, IRR052 and IRR071 fell into the same monophylogenetic group with 100% bootstrap value (Figure 3D). Shimodaira–Hasegawa test showed that the difference of phylogenetic histories inferred from different regions were statistically significant (*p* < 0.001).

When RDP was run to scan the alignment data of the 433 L segments, it was found that another 19 L segments had the same recombination signal as IRR052. Of them, 18 were determined by the incongruent phylogenetic histories of different regions (Figures 3E,F) (*p* < 0.001 of Shimodaira–Hasegawa test).

All these results show that many LASV families with recombinant segment are circulating in Africa. It was noticed that some of recombinant segments were single. At present, we cannot determine whether they are natural or non-natural recombinants due to co-infected samples. To confirm whether they are natural recombinants, further purification and sequencing may be necessary.

The occurrence of recombination requires two viruses to infect a host cell at the same time. Persistent infection may provide more opportunities for such co-infection. Interestingly, previous studies indicated that LASV infection can downregulates its cell-surface receptor, alpha-DG, which inhibits co-infection (Rojek et al., 2007). This downregulation could increase viral diversity, potentially influencing LASV recombination and evolutionary dynamics. Reassortment has been found in this virus (Andersen et al., 2015), suggesting that co-infection of LASV can occur in the same cell. Although LASV is an acute infection in humans, it can cause the persistent infection in rodent host (Walker et al., 1975; Marien et al., 2017). Thus, these recombination events are more likely to occur in *Mastomys natalensis* than in humans. In addition, although HR is a rare event in the virus, interestingly, recombinants are not unusual in the current prevalent LASV. The discovery of these recombinants also suggests that co-infection of LASV might be common in its reservoir host.

Although most recombination breakpoints are randomly distributed in S and L segments, several recombinants have a similar breakpoint near site 3,000 of S segment, including three single sequences and three groups, namely, IRR_030, 0169-ONDO, IRR_ 004, IRR_ 073 group, Barlie-00241 group, and G2906 group. This suggested that this site might be a so-called recombination hotspot. According to the template shift hypothesis of RNA virus recombination, recombination occurred in RNA regions, where RNA could potentially form a secondary structure and play a role in recombination as activator (Nagy and Simon, 1997). This allows the two parental RNAs of different origins to form a complex and thereby forces recombination to occur (Romanova et al., 1986; Tolskaya et al., 1987). We analyzed the secondary structure of 100 nucleotides around this site of recombinant and found that there is a potentially hairpin structure with a minimum free energy of approximately −20 kcal/mol (Supplementary Figure S14). Similar secondary structures have been observed in recombination studies of other viruses. These secondary structures may promote template switching by stalling the RNA polymerase during replication and facilitating the transfer of the polymerase and the nascent nucleic acid molecule onto the acceptor RNA. Consequently, these structures are considered as a factor driving the occurrence of recombination (Chare and Holmes, 2006; Simon-Loriere and Holmes, 2011). These findings further support our hypothesis that RNA secondary structures may play a crucial role in the process of viral recombination. The presence of such secondary structures provides an interaction platform for RNAs of different origins, facilitating template switching and gene recombination.

HR can sometimes play a major role in evolution, emergence, and epidemiology of virus. It has been found that recombination has been associated with the expansion of viral host range (Gibbs and Weiller, 1999), increases in virulence (Khatchikian et al., 1989), the evasion of host immunity (Malim and Emerman, 2001), and the evolution of resistance to antivirals (Nora et al., 2007). In this study, the recombinant rate of S segment was found to be significantly higher than that of L segment, which may be related to the different functions of the proteins encoded by these two segments. The proteins encoded by the S fragment bears the main pressure of host immune selection, which may be one of the reasons for the difference in recombination rates between the two fragments. GP1 and GP2 encoded by S segment are crucial for host cell attachment and virus entry. NP possesses several mechanisms as an anti-innate immune response at the 3’end, and one of them is known as DEDDh motif (Hastie et al., 2012), which aids in immune evasion. We found that all recombinant offspring have the motif. Interestingly, we found that an isolate lacking the motif was located at the ancestral position of one parent lineage (Supplementary Figure S15), suggesting that recombination may have given its offspring the potential to evade host immune pressure.

In conclusion, this study found that HR might be a basic genetic mechanism driving the rapid evolution of LASV. In Africa, LASVs with intrasegmental recombinant have an important responsibility in the outbreak of LF. Given that HR may not be a high probability event in LASV, it may be necessary to clarify whether the high proportion of recombinants in infected people is due to their higher human tropism or there is such a high proportion of recombinants in its rodent reservoirs.

## Data availability statement

The datasets presented in this study can be found in online repositories. The names of the repository/repositories and accession number(s) can be found in the article/Supplementary material.

## Author contributions

C-QH: Conceptualization, Formal analysis, Funding acquisition, Methodology, Resources, Software, Validation, Visualization, Writing – original draft, Writing – review & editing. CK: Data curation, Formal analysis, Investigation, Methodology, Resources, Software, Validation, Visualization, Writing – original draft, Writing – review & editing. MH: Data curation, Formal analysis, Investigation, Methodology, Resources, Software, Validation, Visualization, Writing – review & editing. G-XC: Data curation, Methodology, Software, Writing – review & editing. S-ML: Data curation, Methodology, Software, Writing – review & editing. N-ZD: Conceptualization, Data curation, Funding acquisition, Resources, Writing – review & editing.

## Footnotes

^1^ https://www.who.int/activities/prioritizing-diseases-for-research-and-development-in-emergency-contexts
