# Supplementary material for: Intrasegmental recombination as an evolutionary force of Lassa fever virus
Source: Front Microbiol. 2024 May 20;15:1411537. doi: 10.3389/fmicb.2024.1411537 (PMC11144899; doi:10.3389/fmicb.2024.1411537)
Supplement: Supplementary file 1 [file Presentation_1.pdf]

Supplementary materials

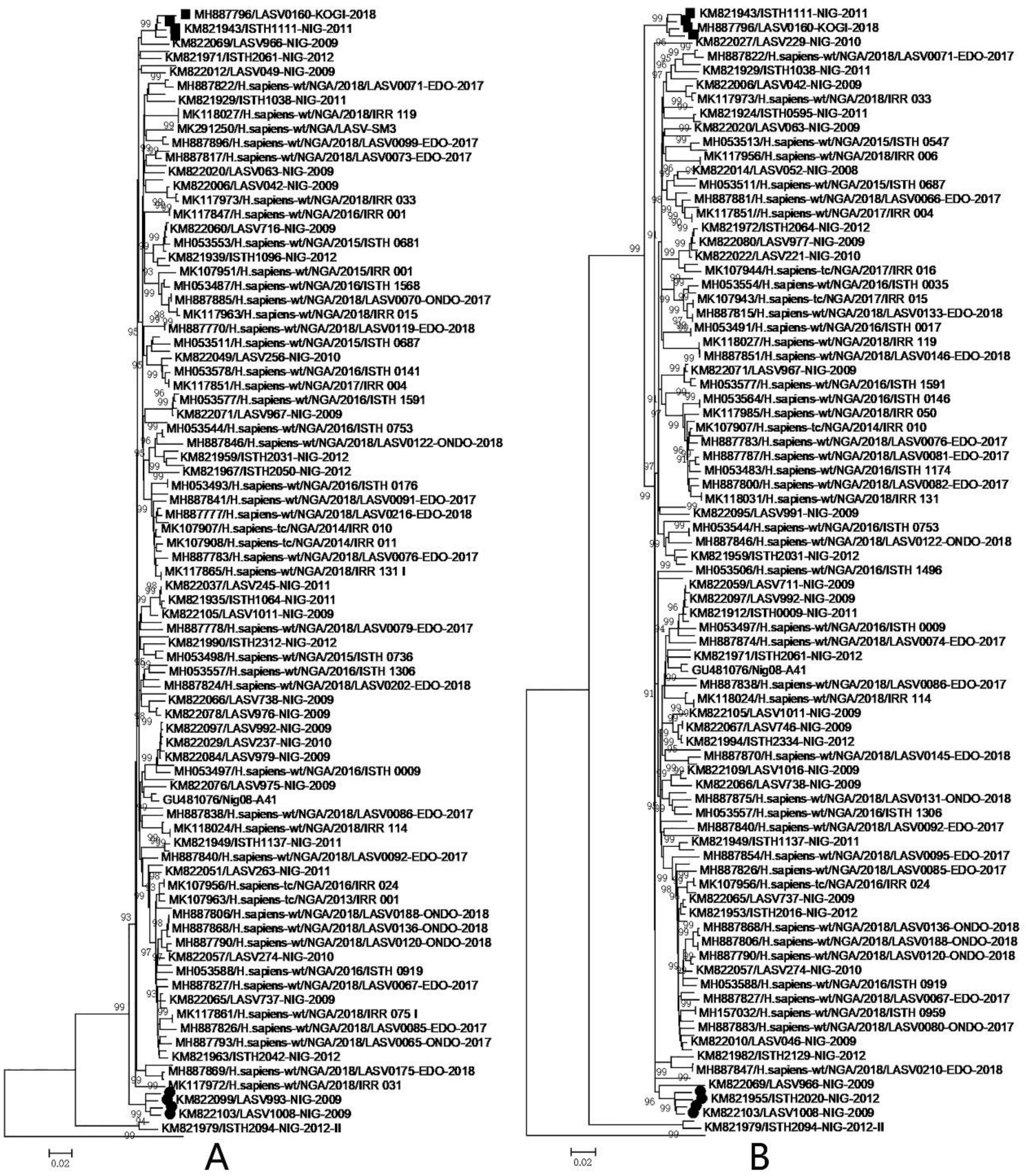

Figure S1. The phylogenetic incongruence of the different regions of the 147 S segment recombinants

A. The phylogenetic history of the recombinants inferred from regions 1-300 and 1369-2169. The recombinants and the minor parent lineage (marked with the black square) constituted a monophylogenetic group. B. The phylogenetic history of the recombinants inferred from regions 301-1368 and 2170-3205. The recombinants and the major parent lineage (marked with the black filled circle) constituted a

monophylogenetic group. The phylogenetic trees were reconstructed using the Neighbor-Joining method with the Tamura 3-parameter substitution model. Evolutionary analyses were conducted in MEGA 11. Square and circle represent the members of the recombinant parent lineages respectively.

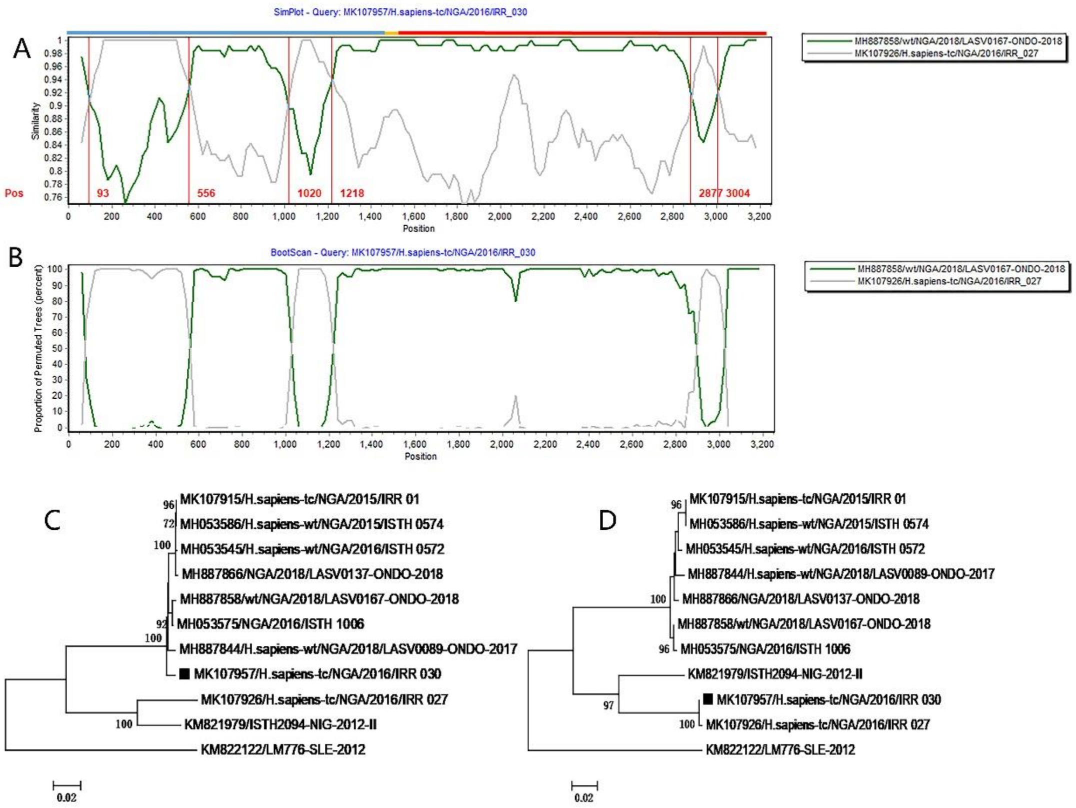

Fig. S2. The evidence for recombination in the S segment of IRR 030

(A) Sequence similarity comparison of S segments of the recombinant IRR 030 and its putative parents IRR 027 and LASV 0167. IRR 030 is used as the query of the comparison. The y-axis indicates the percentage of similarity between IRR 030 and its two putative parents. The vertical lines indicate the recombination breakpoints. Different colors on the bold line represent the different genes in the S segment. Blue, GPC; Yellow, interval; Red, NP. (B) Bootscan plot of S segment sequences of IRR 030 and its putative parents. The y-axis gives the percentage of permutated trees of information sites. (C) and (D) are the phylogenic trees inferred from different regions of S segment. (C) The phylogenic tree inferred from the regions 93-556 + 1020-1218 +2877-3004. (D) The phylogenic tree inferred from the region 1218-2877. Only bootstrap values > 70% are displayed. The Recombinant isolates are marked with "■".

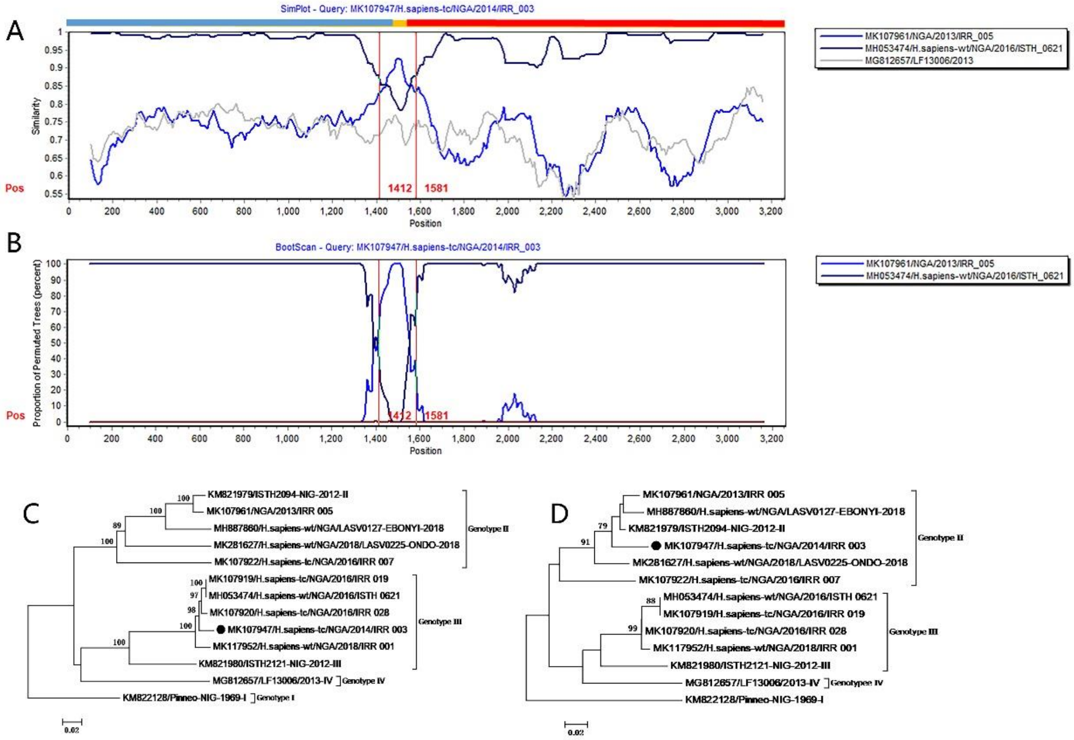

Fig. S3. The evidence for recombination in the S segment of IRR 003

(A) Sequence similarity of S segments of the recombinant IRR 003 and its putative parents ISTH 0621 and IRR 005. IRR 003 is used as the query. The *y*-axis indicates the percentage of similarity between IRR 003 and its two putative parents. Different colors on the bold line represent the different genes in the S segment. Blue, GPC; Yellow, interval; Red, NP. (B) Bootscan plot of S segment sequences of IRR 003 and its putative parents. The y-axis gives the percentage of permutated trees of information sites. The vertical lines indicate the recombination breakpoints. (C) and (D) are the phylogenic trees inferred from different regions of S segment. (C) The phylogenic tree inferred from the regions 1- 1412 +1581-3158. (D) The phylogenic tree inferred from the region 1412-1581. Only bootstrap values > 70% are displayed.

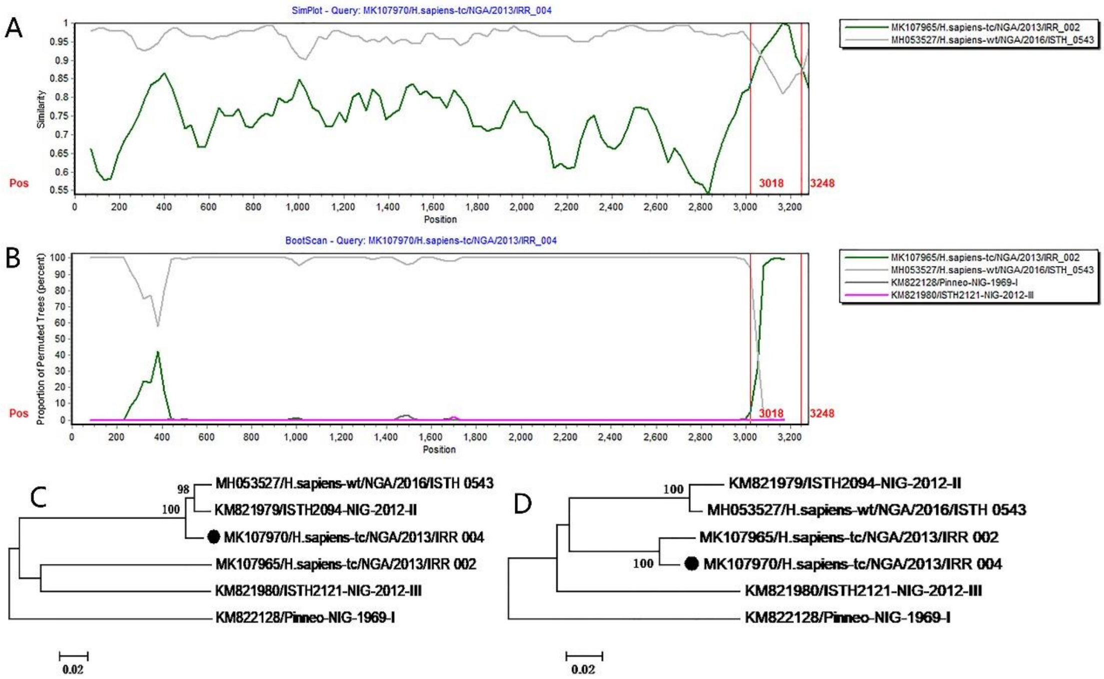

Fig. S4. The evidence for recombination in the S segment of IRR 004

(A) Sequence similarity of S segments of the recombinant IRR 004 and its putative parents ISTH 0543 and IRR 002. IRR 004 is used as the query of the comparison. The *y*-axis indicates the percentage of similarity between IRR 004 and its two putative parents. (B) Bootscan plot of S segment sequences of IRR 004 and its putative parents. The y-axis gives the percentage of permutated trees of information sites. The vertical lines indicate the recombination breakpoints. (C) and (D) are the phylogenic trees inferred from different regions of S segment. (C) The phylogenic tree inferred from the region 1- 3018. (D) The phylogenic tree inferred from the region 3018-3248. Only bootstrap values > 70% are displayed in the trees.

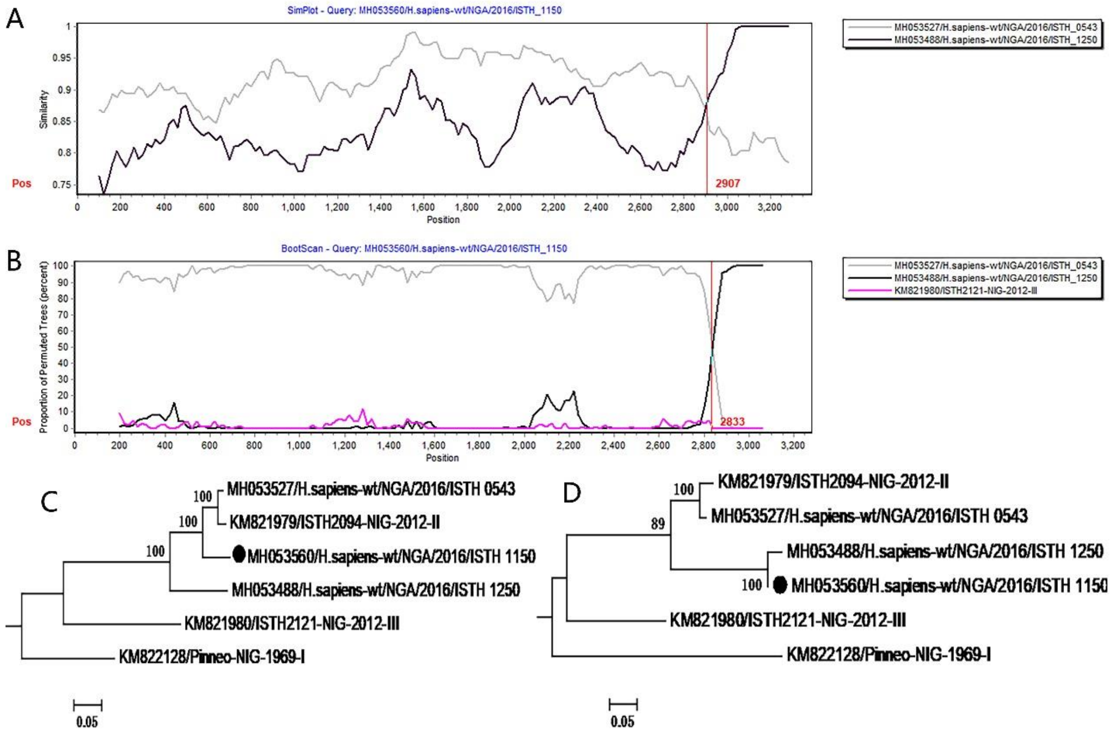

Fig. S5. The evidence for recombination in the S segment of ISTH 1150

(A) Sequence similarity comparison of S segments of the recombinant ISTH 1150 and its putative parents ISTH 1250 and ISTH 0543. ISTH 1150 is used as the query of the comparison. The *y*-axis indicates the percentage of similarity between ISTH 1150 and its putative parents. (B) Bootscan plot of S segment sequences of ISTH 1150 and its putative parents. The y-axis gives the percentage of permutated trees. The vertical line indicates the recombination breakpoint. (C) and (D) are the phylogenic trees inferred from different regions of S segment. (C) The phylogenic tree inferred from the region 1- 2907. (D) The phylogenic tree inferred from the regions 2908-3258. Only bootstrap values > 70% are displayed in the trees.

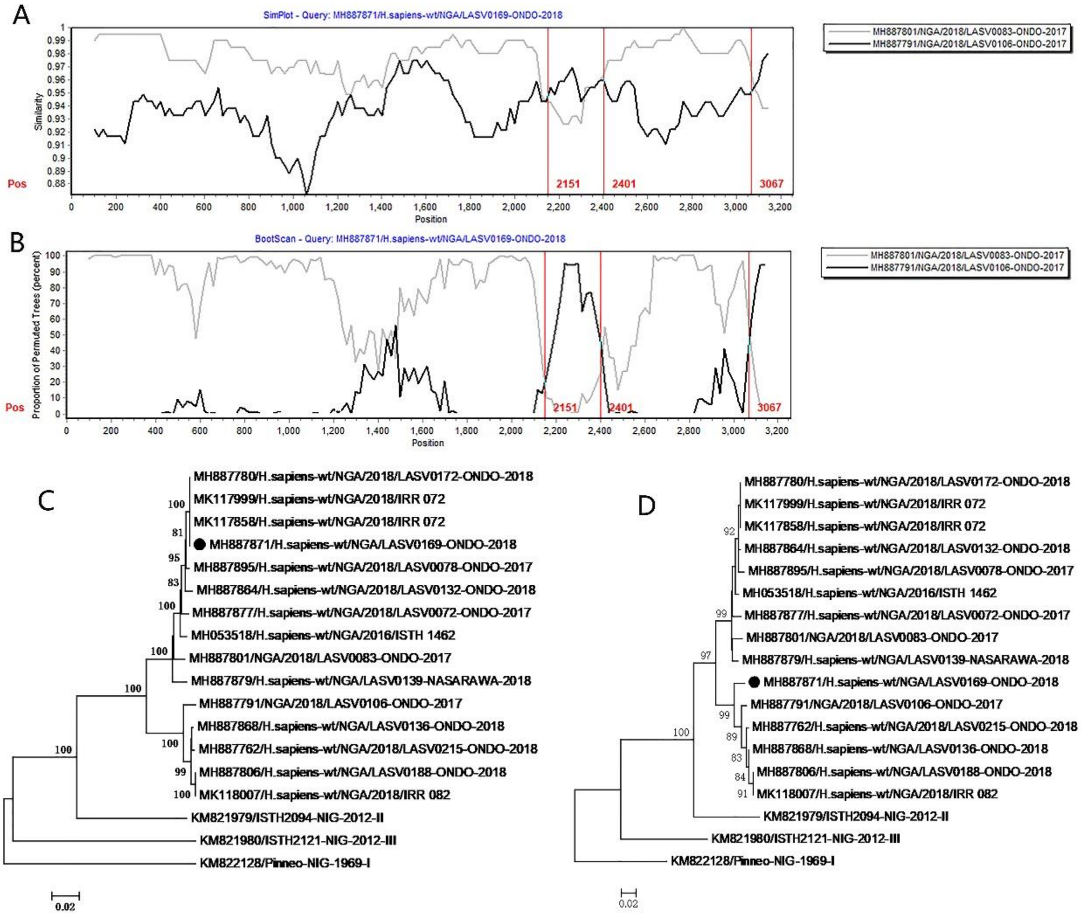

Fig. S6. The evidence for recombination in the S segment of LASV 0169

(A) Sequence similarity comparison of S segments of the recombinant LASV 0169 and its putative parents LASV 0038 and LASV 0106. LASV 0169 is used as the query of the comparison. The *y*-axis indicates the percentage of similarity between LASV 0169 and its putative parents. (B) Bootscan plot of S segment sequences of LASV 0169 and its putative parents. The y-axis gives the percentage of permutated trees of the information sites. The vertical lines indicate the recombination breakpoints. (C) and (D) are the phylogenic trees inferred from different regions of S segment. (C) The phylogenic tree inferred from the region 1- 2151. (D) The phylogenic tree inferred from the regions 2151-2401 + 3067-3258. Only bootstrap values > 70% are displayed in the trees.

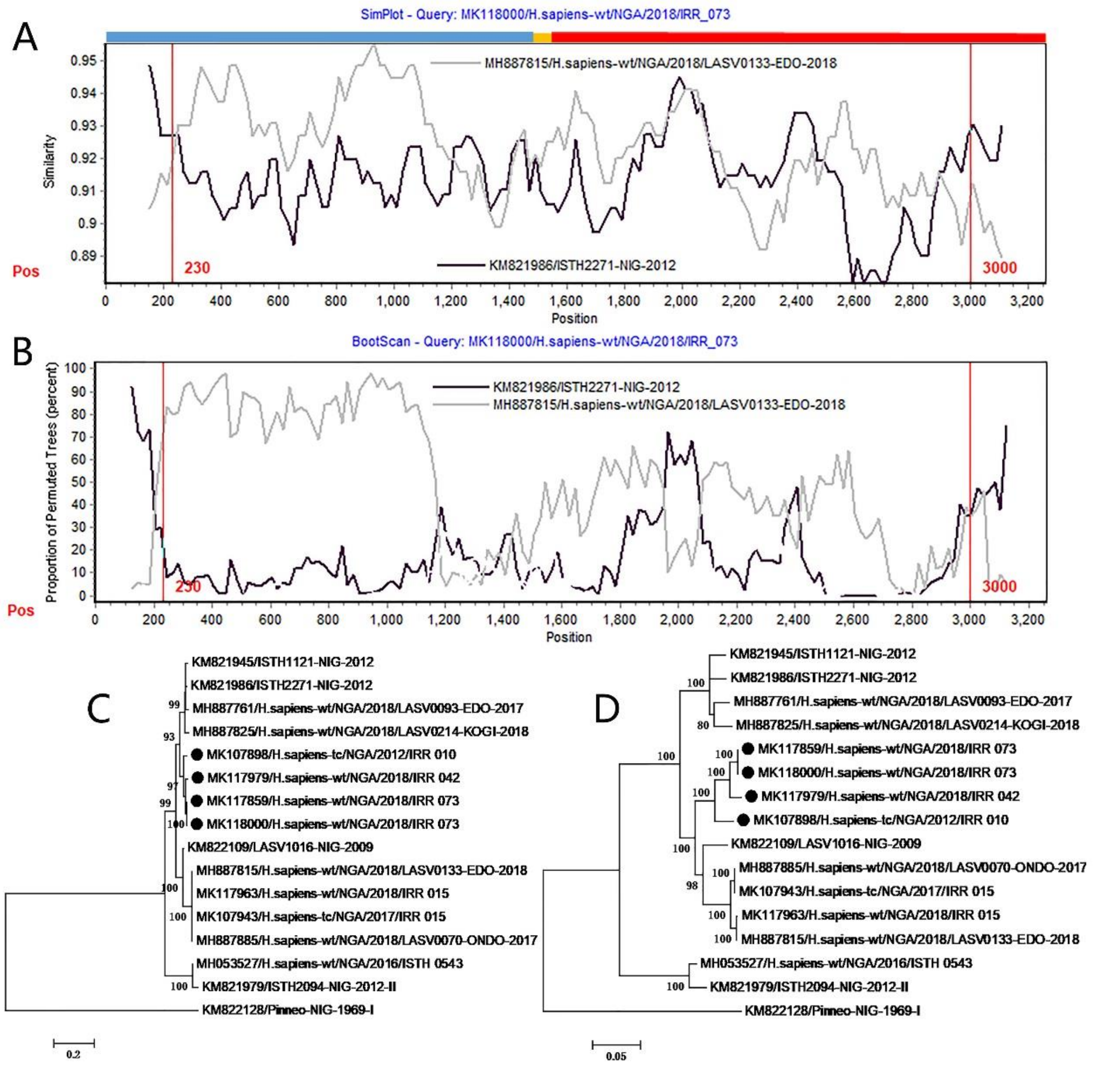

Fig. S7. The evidence for recombination in the S segment of IRR 073 and its sisters

(A) Sequence similarity comparison of S segments of IRR 073 and its putative parents ISTH 2271 and LASV 0133. IRR 073 is used as the query of the comparison. The *y*-axis indicates the percentage of similarity between IRR 073 and its putative parents. Different colors on the bold line represent the different genes in the S segment. Blue, GPC; Yellow, interval; Red, NP. (B) Bootscan plot of S segment sequences of IRR 073 and its putative parents. The y-axis gives the percentage of permutated trees of the information sites. The vertical lines indicate the recombination breakpoints. (C) and (D) are the phylogenic trees inferred from different regions of S segment. (C) The phylogenic tree inferred from the regions 1-230 + 3001-3262. (D) The phylogenic tree inferred from the region 231-3000. Bootstrap values < 70% are hidden in the trees.

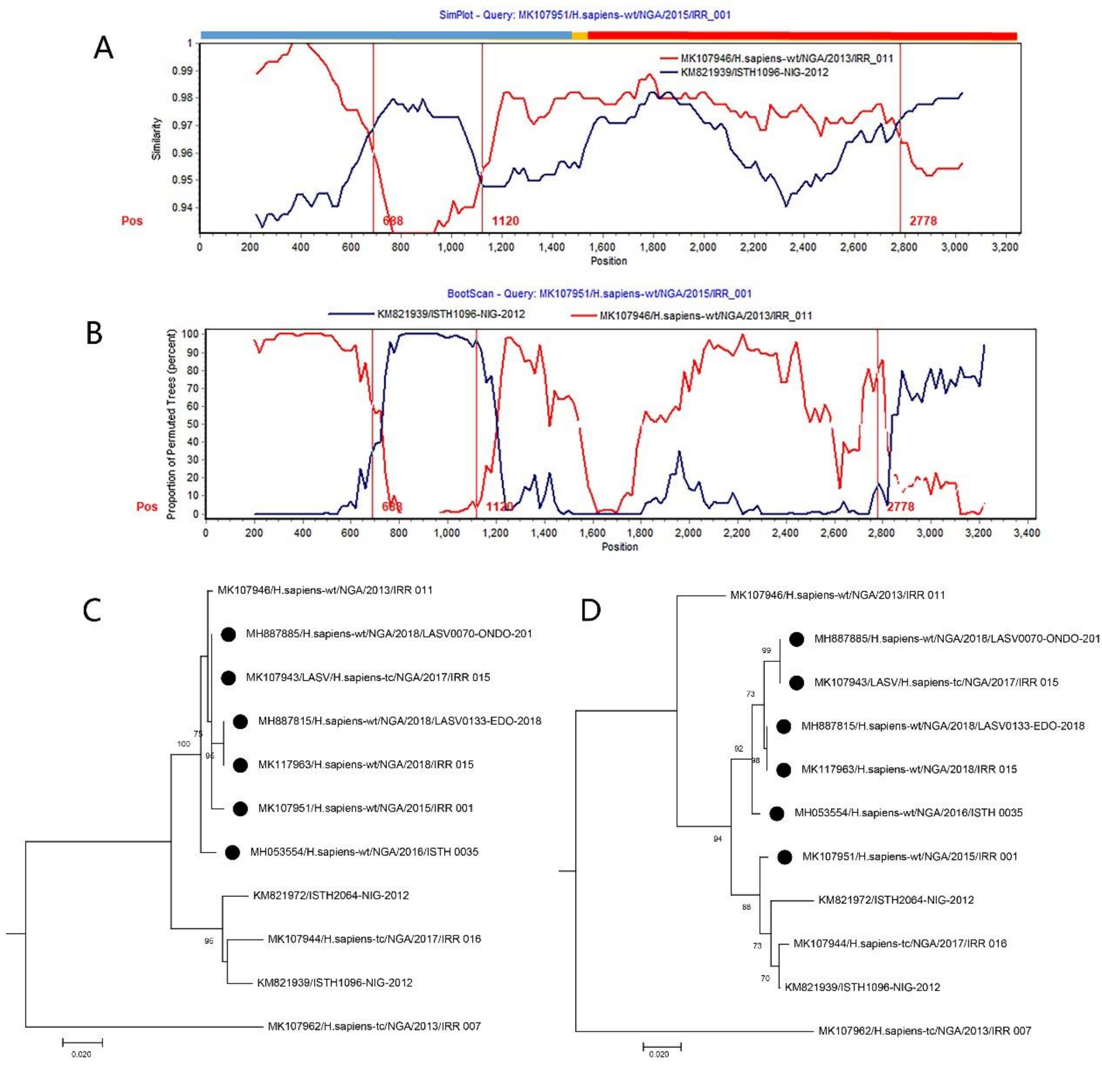

Fig. S8. The evidence for recombination in the S segment of IRR 001 and its sisters

(A) Sequence similarity comparison of S segments of IRR 001 and its putative parents ISTH 0196 and IRR 011. IRR 001 is used as the query of the comparison. The *y*-axis indicates the percentage of similarity between IRR 001 and its putative parents. Different colors on the bold line represent the different genes in the S segment. Blue, GPC; Yellow, interval; Red, NP. (B) Bootscan plot of S segment sequences of IRR 001 and its putative parents. The y-axis gives the percentage of permutated trees of the information sites. The vertical lines indicate the recombination breakpoints. (C) and (D) are the phylogenic trees inferred from different regions of S segment. (C) The phylogenic tree inferred from the regions 1-688 + 1120-2778. (D) The phylogenic tree inferred from the regions 688-1120 +2779-3420. Bootstrap values < 70% are hidden in the trees.

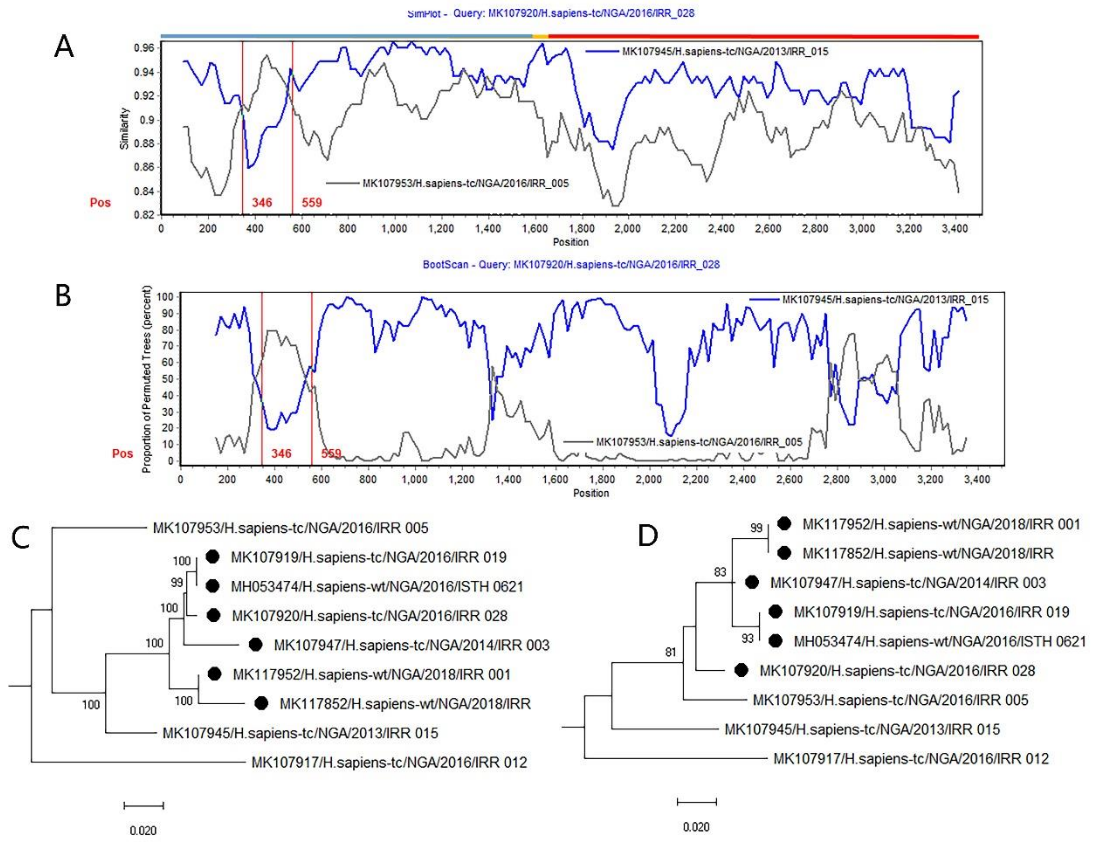

Fig. S9. The evidence for recombination in the S segment of IRR 028 and its sisters (A) Sequence similarity comparison of S segments of IRR 028 and its putative parents IRR 005 and IRR 015. IRR 028 is used as the query of the comparison. The *y*-axis indicates the percentage of similarity between IRR 028 and its putative parents. Different colors on the bold line represent the different genes in the S segment. Blue, GPC; Yellow, interval; Red, NP. (B) Bootscan plot of S segment sequences of IRR 028 and its putative parents. The y-axis gives the percentage of permutated trees of the information sites. The vertical lines indicate the recombination breakpoints. (C) and (D) are the phylogenic trees inferred from different regions of S segment. (C) The phylogenic tree inferred from the regions 1-346 + 559-3400. (D) The phylogenic tree inferred from the region 347-559. Bootstrap values < 70% are hidden in the trees.

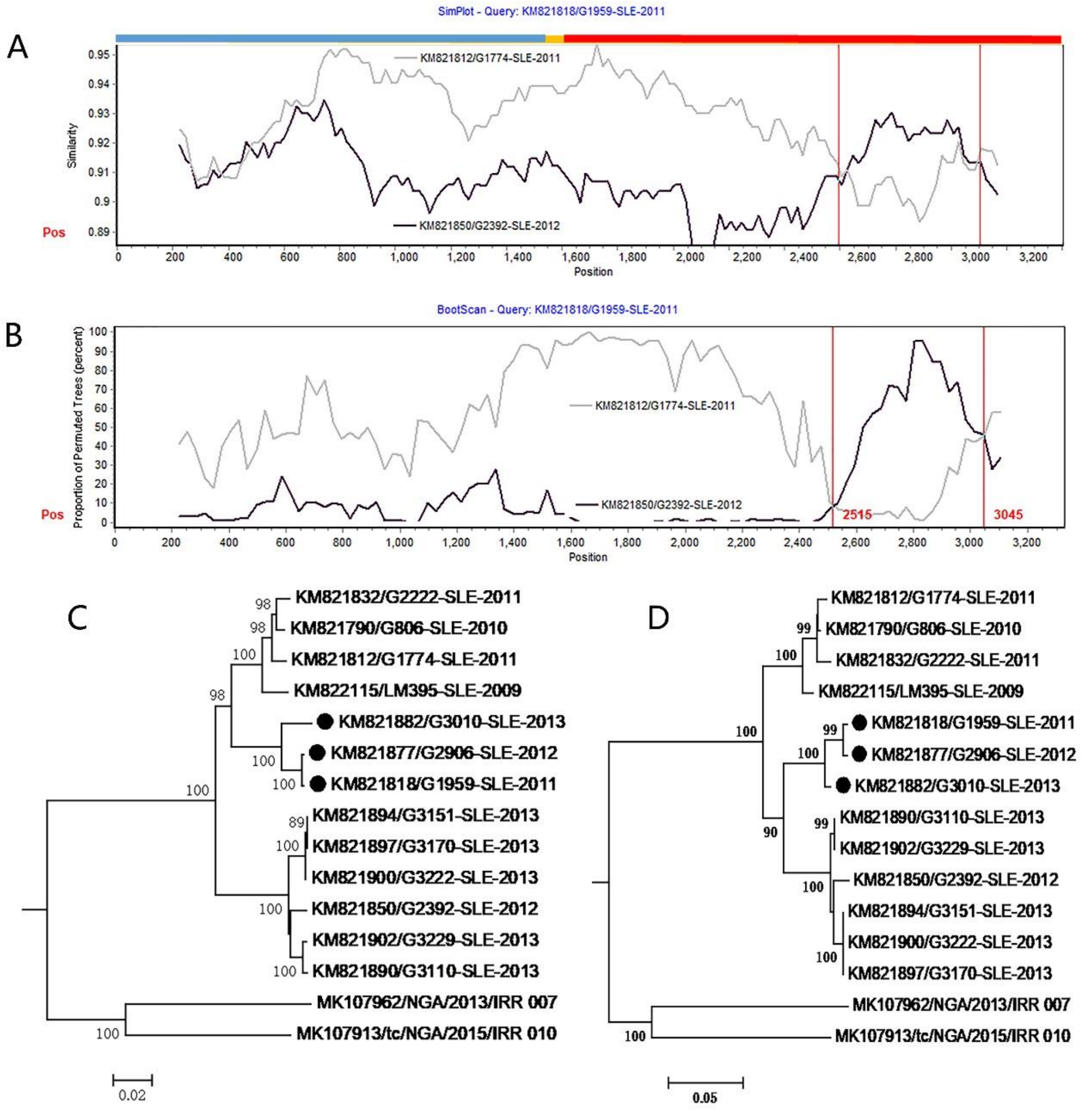

Fig. S10. The evidence for recombination in the S segment of G1959 and its sisters circulating in Sierra Leone

(A) Sequence similarity of S segments of the recombinant G1959 and its putative parents G1774 and G2392. G1959 is used as the query. The y-axis indicates the percentage of similarity between G1959 and its two parents. Different colors on the bold line represent the different genes in the S segment. Blue, GPC; Yellow, interval; Red, NP. (B) Bootscan plot of S segment sequences of G1959 and its putative parents. The y-axis gives the percentage of permutated trees. The vertical lines indicate the recombination breakpoints. (C) and (D) are the phylogenic trees inferred from different regions of S segment. (C) The phylogenic tree inferred from the region 1-2515. (D) The phylogenic tree inferred from the regions 2515-3045. Only bootstrap

values > 70% are displayed. The Recombinant isolates are marked with “●”.

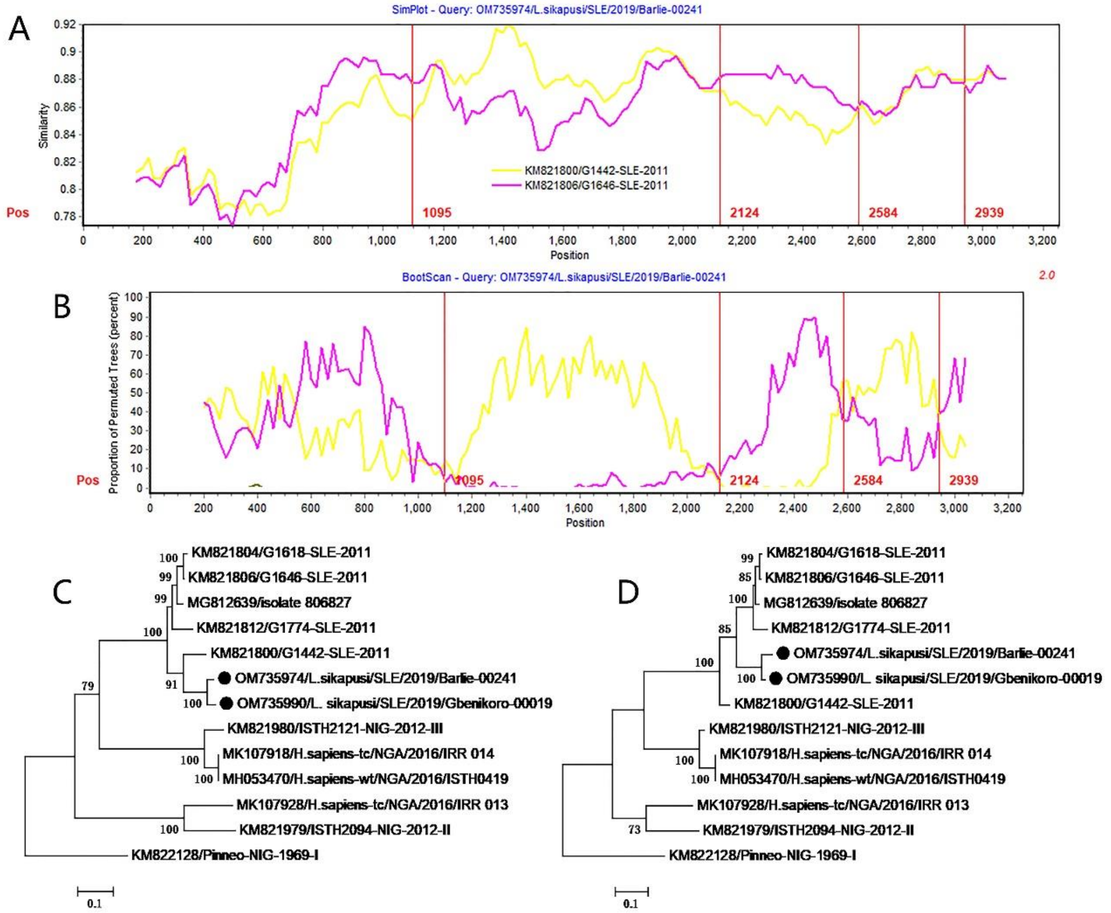

Fig. S11. The evidence for recombination in the S segment of Barlie 00241 and its sister (A) Sequence similarity comparison of S segments of Barlie 00241 and its putative parents G1442 and G1618. Barlie 00241 is used as the query of the comparison. The *y*-axis indicates the percentage of similarity between Barlie 00241 and its putative parents. (B) Bootscan plot of S segment sequences of Barlie 00241 and its putative parents. The y-axis gives the percentage of permutated trees of the information sites. The vertical lines indicate the recombination breakpoints. (C) and (D) are the phylogenic trees inferred from different regions of S segment. (C) The phylogenic tree inferred from the regions 1095-2124 + 2584-2939. (D) The phylogenic tree inferred from the regions 2124-2584 + 2939-3256. Bootstrap values < 70% are hidden in the trees.

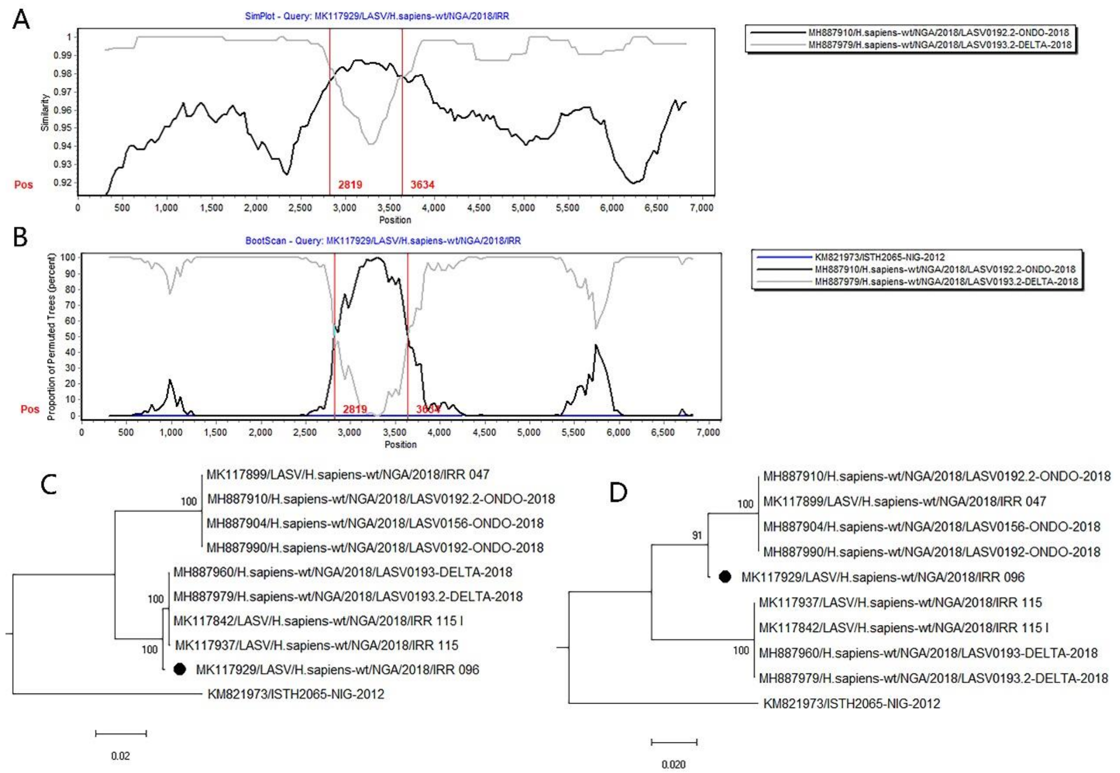

Fig. S12. The evidence for recombination in the L segment of IRR 096

(A) Sequence similarity comparison of L segments of IRR 096 and its putative parents LASV 0193 and LASV 0192. IRR 096 is used as the query of the comparison. The *y*-axis indicates the percentage of L similarity between IRR 096 and its putative parents. (B) Bootscan plot of L segment sequences of IRR 096 and its putative parents. The y-axis gives the percentage of permutated trees of the information sites. The vertical lines indicate the recombination breakpoints. (C) and (D) are the phylogenic trees inferred from different regions of L segment. (C) The phylogenic tree inferred from the regions 1-2819 + 3634-7000. (D) The phylogenic tree inferred from the region 2819-3634. Bootstrap values < 70% are hidden in the trees.

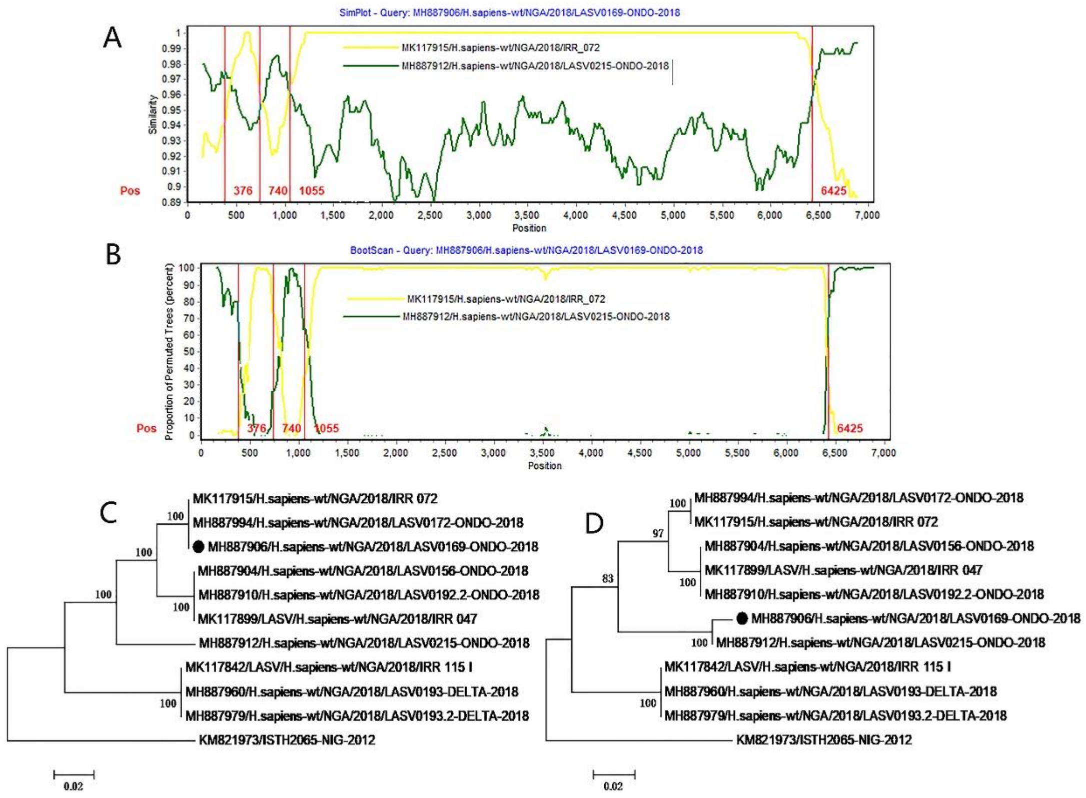

Fig. S13. The evidence for recombination in the L segment of LASV0169

(A) Sequence similarity comparison of L segments of LASV0169 and its putative parents IRR072 and LASV0215. IRR0169 is used as the query of the comparison. The *y*-axis indicates the percentage of L similarity of IRR0169 and its putative parents. (B) Bootscan plot of L segment sequences of IRR0169 and its putative parents. The y-axis gives the percentage of permutated trees. The vertical lines indicate the recombination breakpoints. (C) and (D) are the phylogenic trees inferred from different regions of L segment. (C) The phylogenic tree inferred from the region 1055-6425. (D) The phylogenic tree inferred from the region 6425-7054. Bootstrap values < 70% are hidden in the trees.

A

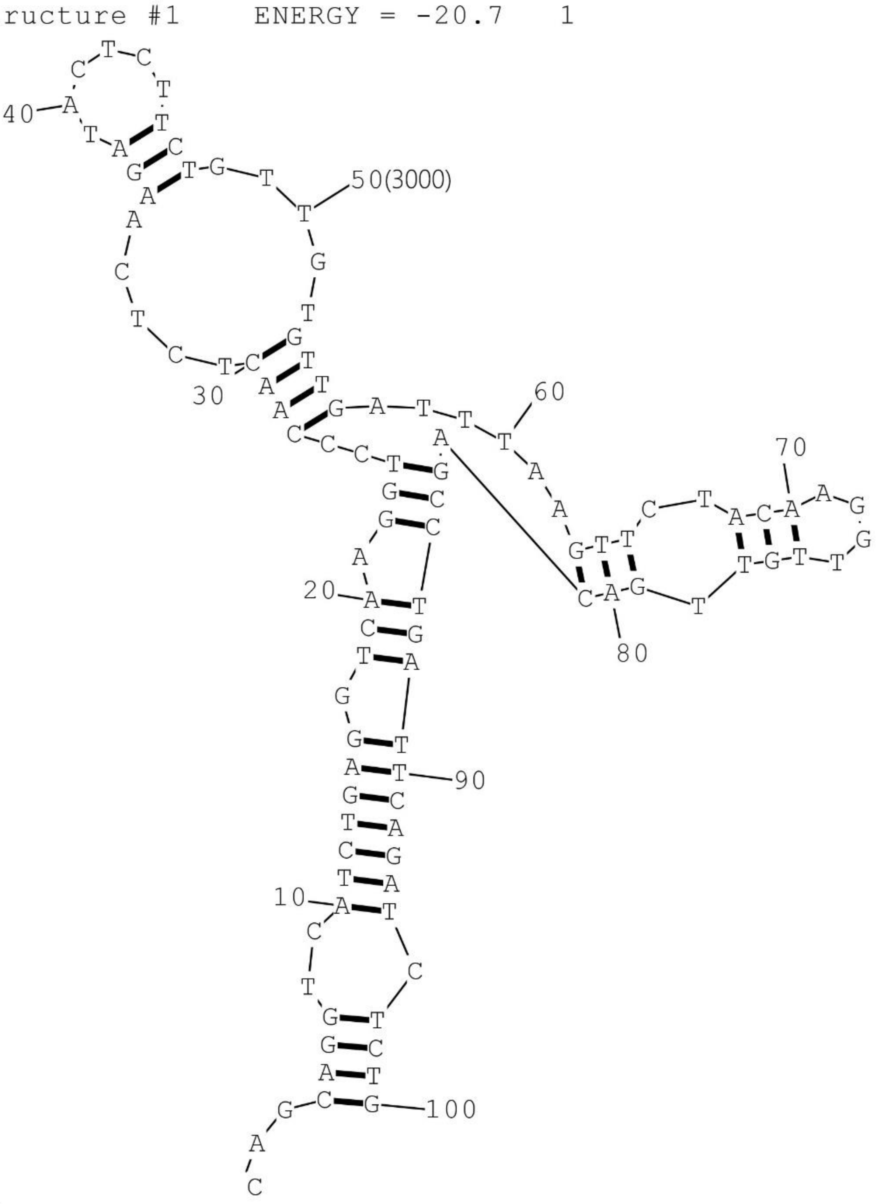
Structure #1 ENERGY = -20.7 1
50(3000)

B

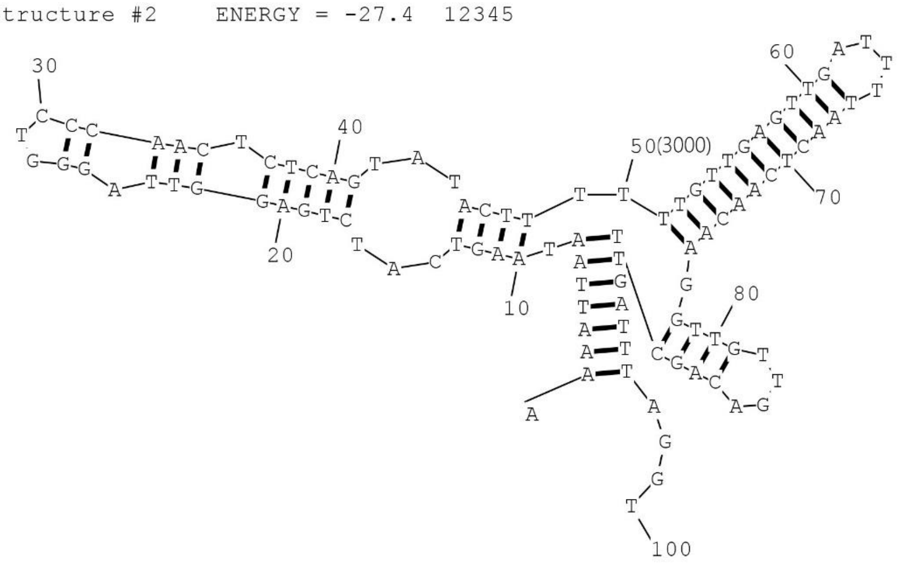
Structure #2 ENERGY = -27.4 12345
50(3000)

Fig. S14. The RNA secondary structure of IRR 073 and G1959 around position 3000 (A) The RNA secondary structure of IRR 073. (C) The RNA secondary structure of G1959.

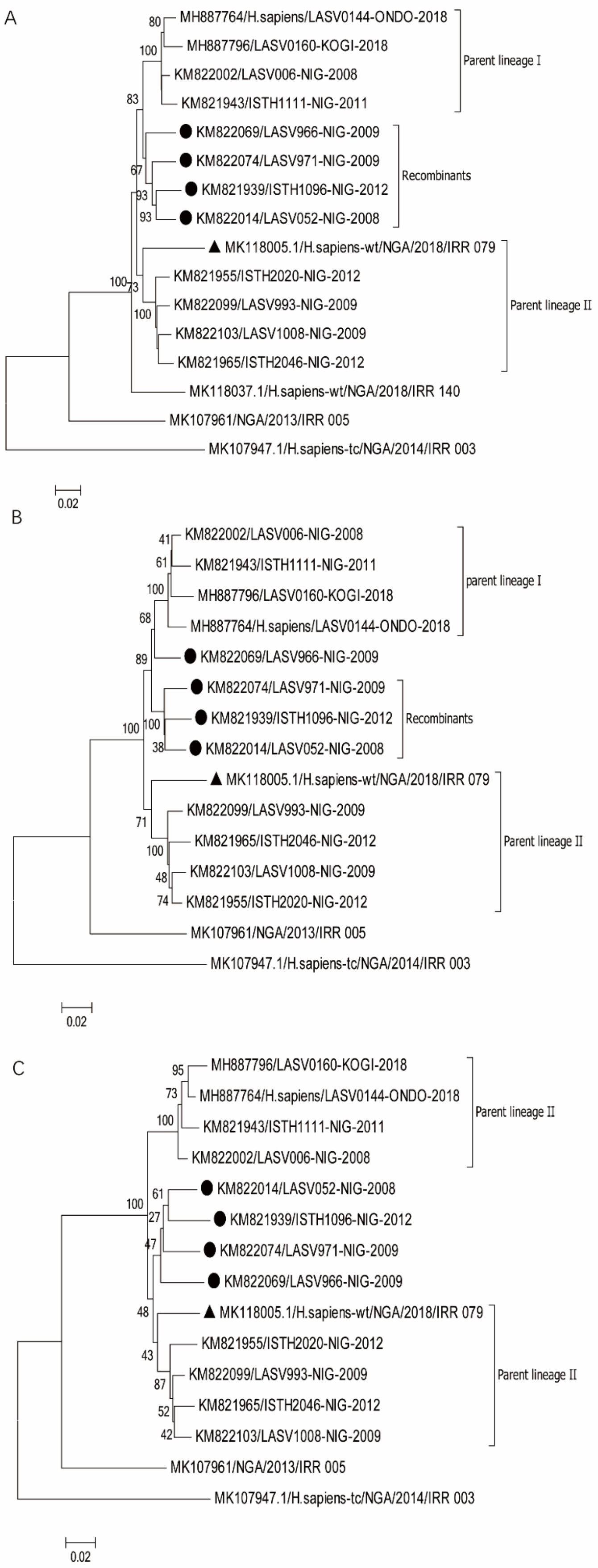

A
MH887764/H.sapiens/LASV0144-ONDO-2018
MH887796/LASV0160-KOGI-2018
KM822002/LASV006-NIG-2008
KM821943/ISTH1111-NIG-2011
Parent lineage I
KM822069/LASV966-NIG-2009
KM822074/LASV971-NIG-2009
KM821939/ISTH1096-NIG-2012
KM822014/LASV052-NIG-2008
Recombinants
MK118005.1/H.sapiens-wt/NGA/2018/IRR 079
KM821955/ISTH2020-NIG-2012
KM822099/LASV993-NIG-2009
KM822103/LASV1008-NIG-2009
KM821965/ISTH2046-NIG-2012
Parent lineage II
MK118037.1/H.sapiens-wt/NGA/2018/IRR 140
MK107961/NGA/2013/IRR 005
MK107947.1/H.sapiens-tc/NGA/2014/IRR 003
80
100
83
67
93
93
100
73
100
0.02
B
KM822002/LASV006-NIG-2008
KM821943/ISTH1111-NIG-2011
MH887796/LASV0160-KOGI-2018
MH887764/H.sapiens/LASV0144-ONDO-2018
parent lineage I
KM822069/LASV966-NIG-2009
KM822074/LASV971-NIG-2009
KM821939/ISTH1096-NIG-2012
KM822014/LASV052-NIG-2008
Recombinants
MK118005.1/H.sapiens-wt/NGA/2018/IRR 079
KM822099/LASV993-NIG-2009
KM821965/ISTH2046-NIG-2012
KM822103/LASV1008-NIG-2009
KM821955/ISTH2020-NIG-2012
Parent lineage II
MK107961/NGA/2013/IRR 005
MK107947.1/H.sapiens-tc/NGA/2014/IRR 003
41
61
100
68
89
100
100
38
71
100
48
74
0.02
C
MH887796/LASV0160-KOGI-2018
MH887764/H.sapiens/LASV0144-ONDO-2018
KM821943/ISTH1111-NIG-2011
KM822002/LASV006-NIG-2008
Parent lineage II
KM822014/LASV052-NIG-2008
KM821939/ISTH1096-NIG-2012
KM822074/LASV971-NIG-2009
KM822069/LASV966-NIG-2009
MK118005.1/H.sapiens-wt/NGA/2018/IRR 079
KM821955/ISTH2020-NIG-2012
KM822099/LASV993-NIG-2009
KM821965/ISTH2046-NIG-2012
KM822103/LASV1008-NIG-2009
Parent lineage II
MK107961/NGA/2013/IRR 005
MK107947.1/H.sapiens-tc/NGA/2014/IRR 003
95
73
100
100
61
27
47
48
43
87
52
42
0.02

Fig. S15. The phylogenic tree inferred from the region NP gene. (A) The phylogenic tree inferred from 930nt near the 3 'end of the NP gene. (B) The phylogenic tree inferred from 930nt near the 3' end of the NP gene. (C) The phylogenic tree inferred from the region from 930 to 1493 near the 3' end of the NP gene. The recombinant representatives were marked with disc. And the isolate without the DEDDh motif was marked with solid triangle.
